# “Unforgettable” – a pictorial essay on anatomy and pathology of the hippocampus

**DOI:** 10.1007/s13244-016-0541-2

**Published:** 2017-01-20

**Authors:** Sven Dekeyzer, Isabelle De Kock, Omid Nikoubashman, Stephanie Vanden Bossche, Ruth Van Eetvelde, Jeroen De Groote, Marjan Acou, Martin Wiesmann, Karel Deblaere, Eric Achten

**Affiliations:** 1 Department of Diagnostic and Interventional Neuroradiology, University Hospital, RWTH Aachen University, Pauwelsstr. 30, 52074 Aachen, Germany; 2 Department of Radiology, University Hospital (UZ) Ghent, De Pintelaan 185, 9000 Ghent, Belgium; 3 Department of Medical Imaging, Onze-Lieve-Vrouw Hospital (OLV) Aalst, Moorselbaan 164, 9300 Aalst, Belgium

**Keywords:** Hippocampus, Epilepsy, Dementia, Herpes simplex encephalitis, MRI

## Abstract

**Abstract:**

The hippocampus is a small but complex anatomical structure that plays an important role in spatial and episodic memory. The hippocampus can be affected by a wide range of congenital variants and degenerative, inflammatory, vascular, tumoral and toxic-metabolic pathologies. Magnetic resonance imaging is the preferred imaging technique for evaluating the hippocampus. The main indications requiring tailored imaging sequences of the hippocampus are medically refractory epilepsy and dementia. The purpose of this pictorial review is threefold: (1) to review the normal anatomy of the hippocampus on MRI; (2) to discuss the optimal imaging strategy for the evaluation of the hippocampus; and (3) to present a pictorial overview of the most common anatomic variants and pathologic conditions affecting the hippocampus.

***Teaching points*:**

*• Knowledge of normal hippocampal anatomy helps recognize anatomic variants and hippocampal pathology.*

*• Refractory epilepsy and dementia are the main indications requiring dedicated hippocampal imaging.*

*• Pathologic conditions centered in and around the hippocampus often have similar imaging features.*

*• Clinical information is often necessary to come to a correct diagnosis or an apt differential.*

## Anatomy, embryology, arterial supply and function

### Normal hippocampal anatomy

**Fig. 1:**
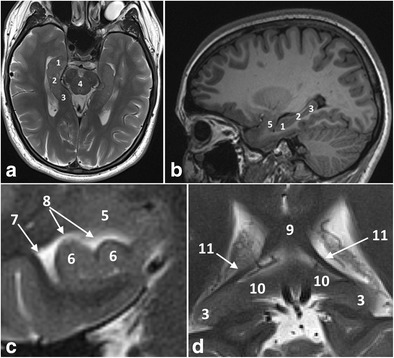
Anatomy of the hippocampal formation on 3-T axial T2 (**a**) and sagittal 3D-MPRAGE images (**b**). Zoomed-in 3-T coronal T2-weighted images at the level of the hippocampal head (**c**) and the hippocampal tail (**d**). The hippocampal body is shown in detail in Fig. 2. *1 = hippocampal head, 2 = hippocampal body, 3 = hippocampal tail, 4 = mesencephalon, 5 = amygdala, 6 = hippocampal digitations, 7 = temporal horn of the lateral ventricle, 8 = uncal recess of the lateral ventricle, 9 = splenium of the corpus callosum, 10 = subsplenial gyri, 11 = crura of the fornices.* To easily recognize the different portions of the hippocampus, we can use the mesencephalon (4). The head (1) is located in front of the mesencephalon, the body (2) can be found at the level of the mesencephalon and the tail (3) is posterior to the mesencephalon. The hippocampal head is the only portion of the hippocampus not covered by the choroid plexus (7). The hippocampal head is separated from the amygdala (5) by the uncal recess of the lateral ventricle (7) and is characterized by small digitations separated by small sulci, the digitationes hippocampi (6). At the level of the hippocampal tail, the fimbriae continue posteriorly as the crux of the fornix (11) that slants upwards towards the splenium of the corpus callosum (9) and the hippocampal tail continues as the subsplenial gyri (10).

**Fig. 2:**
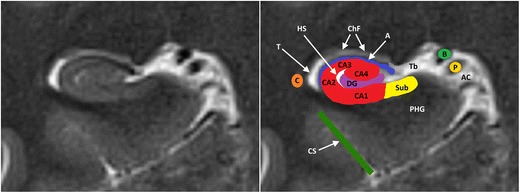
Anatomy of the hippocampal formation at the level of the hippocampal body on 3-T coronal T2. The hippocampal formation consists of the cornu ammonis or hippocampus proper, which can histologically be divided in the four Sommer sectors CA1–CA4, and the dentate gyrus (DG). A small hippocampal cyst (Hs) reflects the location of the largely obliterated hippocampal sulcus. A = alveus, Ac = Ambient cistern, B = basal vein of Rosenthal, C = tail of caudate nucleus, ChF = choroid fissure, CS = collateral suclus, DG = dentate gyrus, P = posterior cerebral artery, PHG = parahippocampal gyrus, Sub = subiculum, T = temporal horn of the lateral ventricle, Tb = transverse fissure of Bichat

The hippocampus is a bilaminar gray matter structure located medially in the temporal lobe that protrudes over the temporal horn of the lateral ventricle and occupies the medial region of its floor (Figs. 1 and 2). The hippocampus consists of two interlocking gray matter folds, the cornu ammonis (or hippocampus proper) and the dentate gyrus. In the axial plane, the hippocampus resembles a seahorse (hence, its name) and it arches around the mesencephalon (hence, the term “mesiotemporal”). In the axial and sagittal plane, it can be divided into three parts: (1) the head or anterior segment; (2) the body or intermediate segment; and (3) the tail or posterior segment. White matter fibres from the hippocampus accumulate on its superior surface to form the alveus. White matter fibres from the alveus then gather medially into thickened bundles as the fimbria, which are continuous posteriorly with the fornix [1].

Based on its cellular composition, the cornu ammonis is divided into four parts, the so-called Sommer’s sectors CA1 to CA4. The cornu ammonis continues inferomedially in the parahippocampal gyrus, a gray matter structure that forms the transition area between the basal and mesial areas of the temporal lobe. The subiculum is the medial and superior edge of the parahippocampal gyrus and its site of union with the cornu ammonis[1].The hippocampus is surrounded by several fissures which are collectively referred to as the perihippocampal fissures. The transverse fissure of Bichat is the lateral extension of the ambient cistern which separates the thalamus superiorly from the parahippocampal gyrus inferiorly. The superolateral extension of the transverse fissure is the choroidal fissure. The inferolateral extension of the transverse fissure is the hippocampal fissure, which extends between the dentate gyrus and the subiculum and is often obliterated and not visible on MRI [1].

### Arterial supply of the hippocampus

**Fig. 3:**
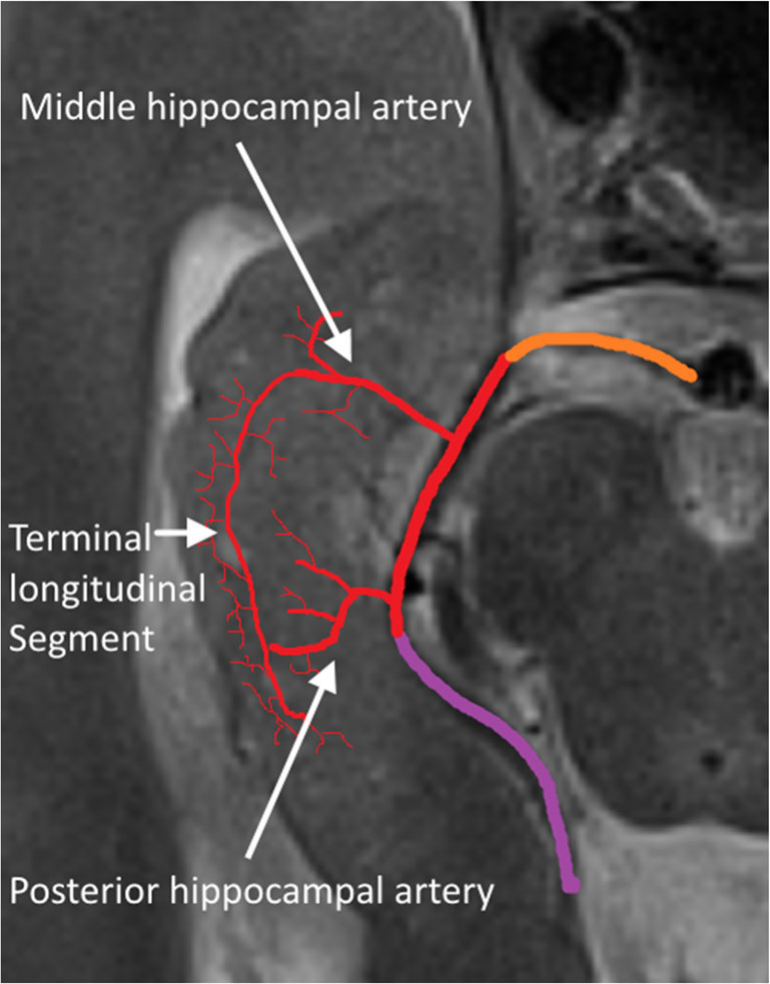
Arterial supply of the hippocampal body and tail. Orange = P1, red = P2 and purple = P3 segment of the posterior cerebral artery. The anterior hippocampal artery is hidden in the uncal sulcus and is shown in Fig. 4

**Fig. 4:**
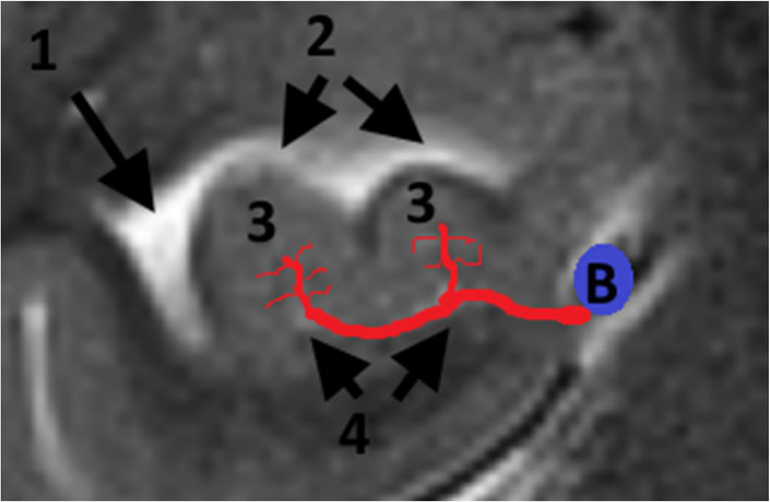
Arterial supply of the hippocampal head. B = basal Rosenthal vein, 1 = temporal horn of the lateral ventricle, 2 = uncal recess of the lateral ventricle, 3 = hippocampal digitations, 4 = uncal sulcus. Both the anterior hippocampal artery, originating from the trunk or branches of the posterior cerebral artery, and the uncal branch of the anterior choroidal artery, dive into the uncal sulcus at the level of the hippocampal head and form anastomoses in the sulci between the hippocampal digitations. Here, only one of both arteries in the uncal sulcus is drawn

Usually, three arteries (or groups of arteries) arising from the main or branches of the posterior cerebral artery vascularize the hippocampus: the anterior, middle and posterior hippocampal arteries. The anterior hippocampal artery supplies the hippocampal head, whereas the middle and posterior hippocampal arteries vascularize the hippocampal body and tail. The middle and posterior hippocampal artery are richly interconnected with another through the so-called longitudinal terminal segments that run parallel to the course of the hippocampal body (Fig. 3). The uncal branch of the anterior choroidal artery is usually anastomosed with the anterior hippocampal artery in the uncal sulcus (Fig. 4) [1].

### Normal hippocampal development

**Fig. 5:**
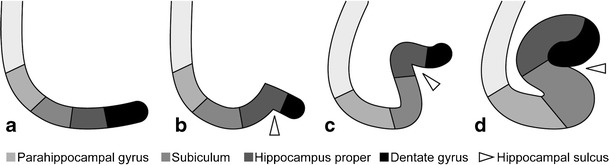
Embryologic development of the hippocampus (image taken from *Atlas klinische Neuroradiologie des Gehirns*, Springer Berlin Heidelberg, 2011, p. 23, by Lin, Wiesmann and Brückmann. With permission of Springer ©)

**Fig. 6:**
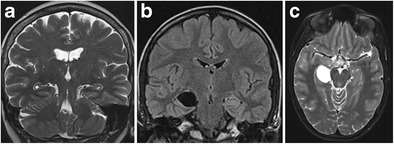
Bilateral sulcal remnant cysts (**a**) and right-sided choroid fissure cyst (**b, c**). Coronal T2 shows small bilateral cysts at the apex of the hippocampal fold between the dentate gyrus and Ammon’s horn (**a**). Coronal FLAIR (**b**) and axial T2-weighted (**c**) images show a space-occupying cystic lesion, iso-intense to cerebrospinal fluid, at the level of the right choroid fissure

The bulk of the cerebral cortex consists of the neocortex, a phylogenetically advanced structure consisting of six cell layers. The mesial temporal structures are formed of more primitive allocortex with three, four or five cell layers and begin their development in early fetal life as a flat cortical plate along the medial wall and floor of the temporal horn (Fig. 5a). Gradual infolding of the various components occurs as a result of expansion of the neocortex and unequal growth of the various hippocampal components (Fig. 5b, c). The infolding occurs around the hippocampal sulcus, a sulcus that develops between the dentate gyrus and cornu ammonis and later shifts to a location between the dentate gyrus and subiculum (Fig. 5d). Eventually, the hippocampal sulcus becomes obliterated, but at times, a residual cavity, a so-called sulcal remnant cyst, may be seen (Fig. 6a).

### Function

The hippocampus plays an important role in spatial and episodic memory. A possible role of the medial temporal lobe in memory formation was first suggested over a century ago by von Bechterew after presenting the neuropathological findings from the brain of a 60-year-old man who had memory problems during the last 20 years of his life and whose brain showed bilateral softening of the gyrus uncinatus and the hippocampal formation [2]. The role of the hippocampus in episodic memory became firmly established in 1957, when Scoville described a case of severe antegrade amnesia following bilateral medial temporal lobe resection in a patient with intractable seizures [3].

## Imaging protocol

The design of the imaging protocol largely depends on the clinical question. Indications that require tailored sequences for optimal visualization of the hippocampus are medically refractory epilepsy and dementia [4, 5].

In patients with medically refractory epilepsy, MRI at 3 T, if available, is preferred, and for the hippocampus, a 1-mm isotropic 3D series with T1W and FLAIR contrast with a good signal-to-noise ratio must be acquired with reconstructions along and perpendicular to the plane of the hippocampi [6, 7]. A tilted coronal T2-weighted (T2W) series should be acquired as well with high resolution (0.5 mm or less in-plane) to evaluate the internal hippocampal structure. If acquisition time is limited, at least coronal IRT1W (1.5 T) or T2W (3 T) images perpendicular to the hippocampal plane should be acquired as extra to a standard screening protocol [8]. Contrast administration is only necessary if findings on the non-enhanced series need further investigation [4].

For an optimal evaluation of the hippocampi in patients with dementia, a 3D T1W sequence with coronal reconstructions perpendicular to the plane of the hippocampi is recommended. If 3D techniques are unavailable, coronal-oblique 2D T1W images can serve as an alternative [5].

## Imaging features of anatomic variants and pathologic entities

### Congenital anomalies

#### Sulcal remnant cysts and choroidal fissure cysts

Sulcal remnant cysts and choroidal fissure cysts are benign cerebral cysts that can occasionally be found at the level of the vestigial hippocampal sulcus and the choroid fissure, respectively (Fig. 6). Sulcal remnant cysts are residual cysts resulting from lack of obliteration of the hippocampal sulcus and are, therefore, most commonly localized laterally, at the apex of the hippocampal fold, between the cornu ammonis and dentate gyrus. The aetiology of choroid fissure cysts is less clear. The tela choroidea is a double layer of the pia mater that invaginates through the choroid fissure to reach the lateral ventricles and to form the choroid plexus. Developmental errors may occur anywhere along the choroid fissure at the time of formation of the primitive choroid plexus, thus forming a cyst, which may be of the neuroepithelial or arachnoid type. On MRI, sulcal remnant cysts and choroid fissure cysts are isointense to cerebrospinal fluid and show no contrast enhancement or restricted diffusion. Both lesions are usually asymptomatic and discovered incidentally. Very rarely can choroid fissure cysts become symptomatic due to mass effect on the surrounding structures [9].

#### Incomplete hippocampal inversion

##### Incomplete hippocampal inversion (IHI)

**Fig. 7:**
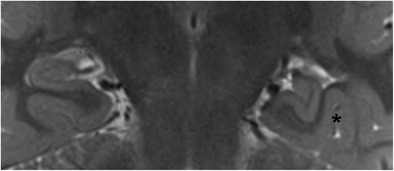
Isolated IHI. Coronal 3-T T2-weighted image in a 23-year-old patient with epilepsy shows a normal hippocampus on the right compared to an incompletely inverted hippocampus with an abnormal rounded or pyramidal shape on the left. The left collateral sulcus (asterisk) has a more vertical orientation and is found lateral of the hippocampal body

**Fig. 8:**
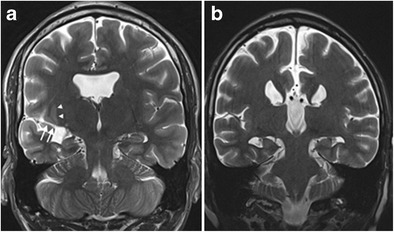
IHI associated with other developmental anomalies. Coronal T2-weighted image in a 22-year-old male patient with mental retardation and epilepsy (**a**) shows incomplete inversion of the right hippocampus (white asterisk at the collateral sulcus) along with a right perisylvian open lip schizencephaly (*white arrows*), right perisylvian subependymal heterotopia (*white arrowheads*) and agenesis of the septum pellucidum. Coronal T2-weighted images in a 25-year-old patient with mental retardation and epilepsy (**b**) shows agenesis of the corpus callosum and an abnormal globular shape of both hippocampi, corresponding to a bilateral incomplete hippocampal inversion

is the result of a failure of hippocampal inversion during normal fetal development. On imaging, the hippocampus has normal signal intensity and volume, but an abnormal globular or pyramidal shape (Figs. 7 and 8). The collateral sulcus has a more vertical orientation than usual and is found lateral from the hippocampal body. IHI is unilateral and left-sided in the majority of cases. The relationship with epilepsy is unclear. IHI has been described in patients with epilepsy and/or severe midline malformations and other developmental malformations, but is also seen in up to 19% of nonepileptic patients without other intracranial abnormalities [10, 11]. In patients with epilepsy, IHI is not considered an epileptogenic lesion per se, but rather a sign of a more widespread disorder of cerebral development that may affect other parts of the brain leading to epilepsy.

### Degenerative diseases

#### Hippocampal calcifications

**Fig. 9:**
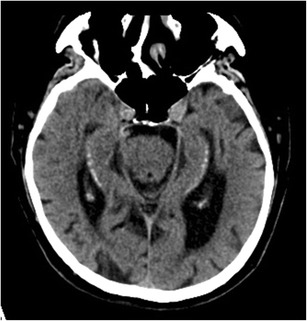
Hippocampal calcifications. Non-enhanced axial CT images angulated parallel to the hippocampal body show bilateral symmetrical calcifications lateral in the hippocampal bodies as a coincidental finding in a 69-year-old female patient with dysarthria

Hippocampal calcifications are a frequent incidental finding on CT (Fig. 9). Their prevalence increases with age and they are described in up to 21.7% of the population over 50 years of age. Hippocampal calcifications are not associated with neurodegenerative diseases. Their pathologic significance is unclear, but they most likely reflect the latter stages of vascular fibrosis [12].

#### Mesial temporal sclerosis

The most common cause of medically intractable partial complex epilepsy in adults is mesial temporal sclerosis (MTS). MTS is characterized pathologically by hippocampal gliosis and neuronal loss. The pathophysiology of MTS is not completely understood. One theory is that early or prolonged febrile seizures damage the hippocampus in genetically susceptible patients. However, the difference between cause and effect is not clear, as it is also conceivable that a child may have prolonged febrile seizures due to MTS secondary to a prenatal/perinatal insult or genetic predisposition [13, 14].

**Fig. 10:**
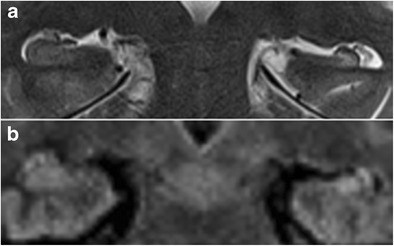
Left-sided mesial temporal sclerosis. 3 T coronal T2-weighted (**a**) and FLAIR images (**b**) in a 43-year-old patient with medically refractory epilepsy show volume loss, increased signal intensity and blurring of the internal structure of the left hippocampus, compatible with mesial temporal sclerosis

Neuronal cell loss and gliosis result in the MRI findings of hippocampal atrophy with increased T2/FLAIR signal intensity (Fig. 10). Other secondary signs can be present such as: (1) loss of the internal architecture of the hippocampus; (2) loss of hippocampal head digitations; and (3) dilatation of the ipsilateral temporal horn. Because of physiologic interconnections between the hippocampus and other parts of the brain, secondary signs can also involve other structures such as: (1) increased signal intensity and/or atrophy of the ipsilateral amygdala; (2) atrophy of the ipsilateral mammillary body; (3) atrophy of the ipsilateral fornix; (4) atrophy of the contralateral cerebellar hemisphere; and (5) atrophy of the ipsilateral entorhinal area [15–17].

**Fig. 11:**
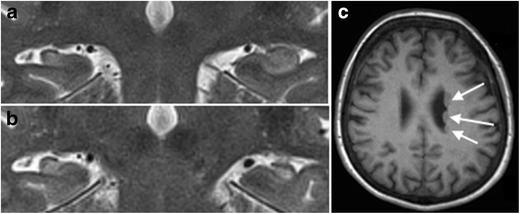
Bilateral hippocampal sclerosis and dual pathology. 3-T coronal T2-weighted image in a 35-year-old patient with medically refractory epilepsy shows volume loss and increased signal intensity of the right hippocampus, compatible with mesial temporal sclerosis (**a**). The left side the hippocampal volume looks normal, but T2 signal intensity is slightly increased in the CA1 and CA2 sectors and there is blurring of the internal structure (**a**). On follow-up MRI performed 3 years later, there is clear bilateral hippocampal sclerosis with atrophy and increased T2 signal intensity of both hippocampi (**b**). Axially reconstructed 3D MPRAGE images (**c**) show multiple periventricular heterotopias (*white arrows*) along the lateral wall of the left lateral ventricle

Identification of MTS is important as surgery is often the only treatment option with a good outcome. Regarding the radiological pre-surgical workout, it is important to note that MTS can be bilateral in up to 3–10% of cases although symptomatology may be unilateral [18], and that dual pathology, the existence of a second epileptogenic lesion, can be present in up to 15% of cases (Fig. 11) [19].

#### Alzheimer’s disease and other dementias

##### Alzheimer’s disease

Alzheimer’s disease (AD) is a progressive neurodegenerative disorder that is characterised pathologically by an accumulation of senile plaques and neuritic tangles. It typically begins in the transenthorinal regions and spreads to the hippocampal cortex and mesial temporal lobes before progressing to the temporal lobes, basal forebrain and the rest of the brain.

**Fig. 12:**
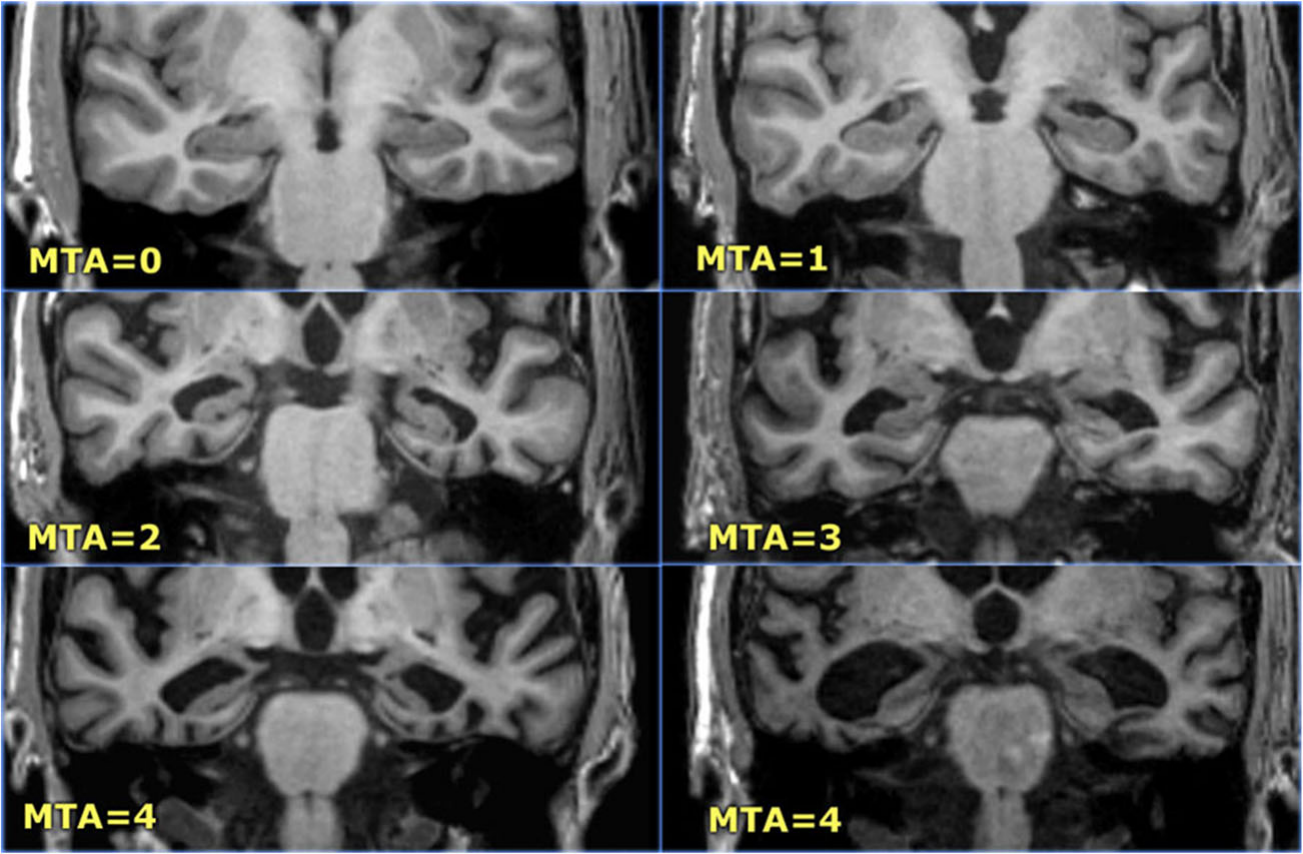
The Scheltens mesial temporal atrophy scale in coronal 3D-MPRAGE images (images from the Radiology Assistant website with permission – http://www.radiologyassistant.nl)

**Fig. 13:**
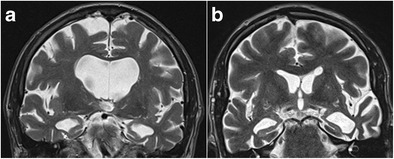
Alzheimer’s disease (**a**) and frontotemporal dementia (**b**). 3-T Coronal T2-weighted images in an 84-year-old patient with clinically advanced sporadic Alzheimer dementia show pronounced mesial temporal atrophy corresponding to an MTA score of 4 (**a**). In comparison, coronal T2-weighted images in a 58-year-old patient with a semantic variant of frontotemporal dementia also shows pronounced bilateral mesiotemporal atrophy (MTA score 4), along with asymmetric pronounced cortical atrophy of the left temporal neocortex (**b**)

Over the past decades, the role of imaging in dementia has shifted from merely excluding possible treatable causes of dementia (e.g. tumour or subdural hematoma) to the identification of certain positive disease markers. In this regard, the mesial temporal atrophy (MTA) score, a radiologic measure for the degree of hippocampal atrophy, has proven to be predictive of progression from mild cognitive impairment to AD [20] and can identify patients with AD with an accuracy and sensitivity of approximately 85% compared to healthy controls [21]. The MTA score is based on four points and takes into account the width of the temporal horn of the lateral ventricle, the width of the choroid fissure and the hippocampal volume (Table 1, Fig. 12) [20]. For subjects younger than 75 years, an MTA score of 2 or more is considered abnormal, while for subjects older than 75 years, an MTA score of 3 or more is considered abnormal [21]. Its usefulness for the distinction of AD from other dementia syndromes is limited however, as MTA not only occurs in AD, but can be seen in other dementia syndromes as well (Fig. 12) [22]. In this regard, it is important to note that the combined visual assessment of mesial temporal atrophy and parietal atrophy has proven to not only increase the sensitivity of MRI in identifying patients with AD, but also increase the specificity of MRI in discriminating AD from other dementia syndromes [23] (Fig. 13; Table 2).

**Table 1:** Visual rating scale of mesial temporal atrophy. N = normal; ↑ = increase; ↓ = decrease

*MTA Score*	Width of choroid fissure	Width of temporal horn	Height of hippocampal formation
*0*	N	N	N
*1*	↑	N	N
*2*	↑↑	↑	↓
*3*	↑↑↑	↑↑	↓↓
*4*	↑↑↑	↑↑↑	↓↓↓

**Table 2:** Overview of the most common variants and pathologic entities centred in and around the hippocampus

	Clinical characteristics	Imaging characteristics	Differential diagnosis
Sulcal remnant cysts	Asymptomatic	CSF iso-intense cyst in the hippocampal fold	/
Choroid fissure cysts	Generally asymptomatic, rarely seizures or symptoms due to mass effect when large	CSF iso-intense cyst in the choroid fissure	/
Incomplete hippocampalinversion	Observed in asymptomatic patients and patients with epilepsy, not considered an epileptogenic focus	Globular or pyramidal shape of the hippocampus with normal signal intensity, usually unilateral and left-sided	/
Alzheimer’s disease	Gradual cognitive decline, usually starts with short-term memory problems	Gradual bilateral volume-loss of the hippocampus and associated mesiotemoral cortex.	Other dementia syndromes, asymmetric temporal atrophy in frontotemporal dementia
Mesial temporal sclerosis	Complex partial epilepsy	Volumeloss and increased T2 signal intensity of the hippocampus with blurring of the internal structure, bilateral in up to 15%.	/
Limbic encephalitis	Subacute onset of confusion, seizures, amnesia, behavioural changes, etc.	Uni- or bilateral mesiotemporal cortical swelling and increased signal intensity, diffusion restriction and patchy enhancement possible, evolves to atrophy.	HSV encephalitis, seizure-induced abnormalities
Herpes simplex encephalitis	Acute onset of fever, headache, seizures, hallucination, personality changes, etc.	Usually starts with unilateral mesiotemporal cortical swelling and increased T2 signal intensity, generally spreads bilaterally. Diffusion restriction, gyral enhancement and petechial hemorrhages possible.	Early stage: limbic encephalitis, seizure-induced abnormalities
Ganglioglioma	Complex partial epilepsy	Typically (40%) well-defined cortical/corticosubcortical cystic mass with an enhancing nodule. Solid T2-hyperintense mass with variable enhancement also possible. Calcifications in 30%.	DNET
DNET	Complex partial epilepsy	Typially multicystic cortical/corticosubcortical lesion with peripheral FLAIR hyperintensity (bright rim sign). Enhancement in 30%, calcifications in < 20%.	Ganglioglioma
Transient global amnesia	Retrograde amnesia lasting < 24 hours	One or more uni- or bilateral intrahippocampal punctiform diffusion-restrictive foci.	More extensive in acute arterial ischemic stroke, generally with other infarcted areas in involved vascular territories
Acute arterial ischemic stroke	Depends on the extent of the infarction and the vascular territory involved	T2-FLAIR hyperintensity and diffusionrestriction of the hippocampal head in anterior choroidal artery infarction and variable invovement of the entire hippocampus in posterior cerebral artery infarction, generally with other areas of infarction in the involved vascular territories.	/
Seizure-induced abnormalities	Recent seizure, postictal state or status epilepticus	FLAIR hyperintensity with or without diffusion restriction of the hippocampus	Limbic encephalitis, HSV encephalitis, infarction

### Infection and inflammation

#### Herpes simplex encephalitis

The hippocampus can also be affected by infectious and inflammatory conditions, the best known of which is herpes simplex encephalitis (HSE). HSE is almost always caused by the HSV type 1 virus, except in neonates where type 2 predominates. The precise sequence of events leading to non-neonatal HSE is unclear. It is often assumed that HSE is caused by reactivation of HSV in the trigeminal ganglion. An alternative hypothesis is that HSE is a primary infection rather than virus reactivation, with the olfactory system as the most probable port of entry [24]. This route of infection would better explain the typical distribution of HSE lesions in the limbic system and the insular cortex.

**Fig. 14:**
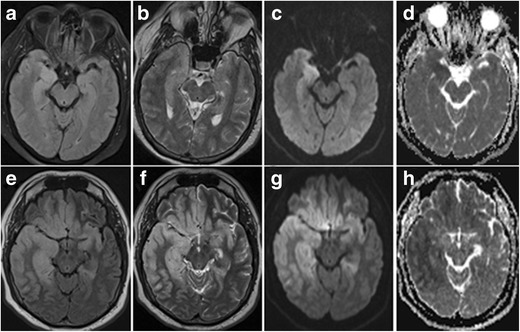
Early stage (**a–d**) versus more advanced (**e–h**) herpes encephalitis. 44-year-old male patient with fever, headache and acute epileptic seizures. 1.5-T axial T2W (**a**), FLAIR (**b**) and diffusion-weighted (**c**) images with an ADC map (**d**) show subtle T2-FLAIR hyperintensity in the right amygdala and hippocampus with diffusion restriction. Follow-up MRI performed one week later (**e–h**) shows more pronounced T2-hyperintense bilateral frontotemporal cortical oedema and diffusion restriction, more pronounced in the right hemisphere. PCR was positive for HSV

**Fig. 15:**
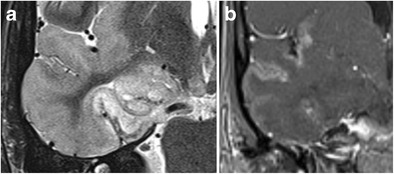
Herpes encephalitis. On coronal T2-weighted images (**a**) extensive corticosubcortical oedema is seen in the right temporal and insular lobe. Notice the presence of cortical T2-hypointense abnormalities in the right hippocampus and along the right collateral sulcus, denoting small petechial haemorrhages. On contrast, enhanced T1-weighted images (**b**), there is extensive right temporal and insular gyral enhancement

Symptoms reflect the propensity to involve the inferomedial frontal and temporal lobes with acute onset of hallucinations, seizures, personality changes and aphasia. HSE primarily affects neurons, which is reflected by preferential cortical involvement on MRI. On MRI, cortical abnormalities are noted as ill-defined areas of high T2/FLAIR signal intensity, usually beginning unilaterally but progressing to become bilateral. Diffusion restriction is not always present, but can be one of the earliest signs of HSE (Fig. 14) [25, 26]. Gyral enhancement and petechial haemorrhages may also be present (Fig. 15).

HSE has a bad prognosis and quick antiviral treatment is mandatory. The mortality rate of HSE exceeds 70% in patients who had no or incomplete treatment and fewer than 3% of all patients return to normal function after recovering from their illness [27].

#### Limbic encephalitis

##### Limbic encephalitis (LE)

is an autoimmune-mediated paraneoplastic or non-paraneoplastic syndrome characterized by the subacute onset of confusion, anterograde amnesia, temporal lobe seizures and behavioural changes. Neuronal antibodies associated with LE can be classified into two groups according to the location of the antigen: inside the neuron or in the cell membrane [28]. The antineuronal antibodies associated with LE are anti-HU, anti-CV2, anti-Ma2 and anti-amphiphysin. These antibodies are generally associated with neoplasia, specifically with small cell lung carcinoma (SCLC), breast carcinoma, testicular tumours and ovarian teratomas. The antibodies against neuronal surface antigens associated with LE include voltage-gated potassium channels (VGKC), AMPA and γ-aminobutyric acid (GABA) and are often non-paraneoplastic.

**Fig. 16:**
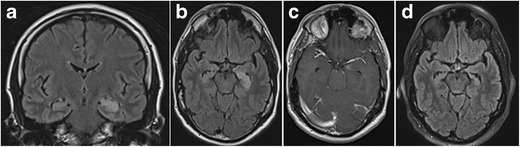
Limbic encephalitis. 1.5-T coronal FLAIR (**a**), axial FLAIR (**b**) and contrast-enhanced T1W images (**c**) show T2-FLAIR-hyperintense swelling of the left hippocampus and discrete patchy enhancement in the left amygdala. On axial FLAIR on follow-up MRI performed 6 months later (**d**), the signal abnormalities have disappeared and there is volume loss of the left hippocampus with loss of the normal hippocampal digitations of the hippocampal head

On MRI, LE manifests with uni- or bilateral swelling and varying degrees of high T2/FLAIR signal intensity in the mesial temporal lobe. Patchy enhancement and restricted diffusion can be seen. These changes may eventually evolve to medial temporal lobe atrophy and increased T2/FLAIR signal intensity in the hippocampus due to gliosis (Fig. 16). Imaging findings can be subtle early in the course of the disease, however, and can even remain normal in a subset of cases [29].

### Tumours

**Fig. 17:**
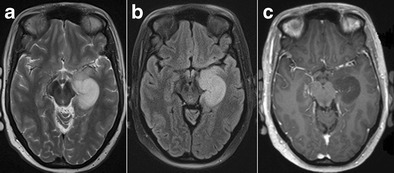
Low-grade glioma. 1.5-T axial FLAIR (**a**), T2W (**b**) and contrast-enhanced T1W images (**c**) show a T2-hyperintense, T1-hypointense, non-contrast-enhancing infiltrative mass in the left hippocampus, corresponding to a pathologically proven glioma

**Fig. 18:**
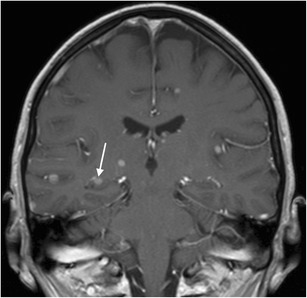
Cerebral metastasis. 1.5-T coronal contrast-enhanced T1W images show multiple bilateral nodular contrast-enhancing lesions, including one in the right hippocampal body (*white arrow*). This was a brain metastasized bronchial carcinoma

**Fig. 19:**
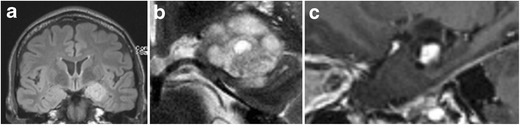
Ganglioglioma. Coronal FLAIR (**a**), zoomed-in coronal T2 (**b**) and zoomed-in sagittal contrast-enhanced 3D-MPRAGE (**c**) show a lobulated T2-FLAIR hyperintense space-occupying lesion in the left amygdala with focal infiltration of the left hippocampal head. The lesion contains a small central cystic (**b**) as well as a nodular contrast-enhancing component (**c**)

**Fig. 20:**
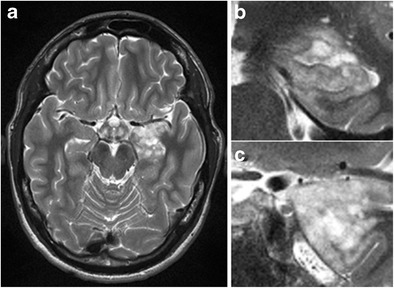
DNET. Axial T2 (**a**) and zoomed-in coronal T2 at the level of the hippocampal head (**b**) and amygdala (**c**) show a multicystic non-enhancing (images not shown) lesion with a bubbly appearance in the cortex and subcortical white matter of the left mesiotemporal lobe infiltrating the left amygdala and hippocampal head

Theoretically, any primary or secondary brain tumour can affect the hippocampus (Fig. 17 and 18), but a specific subgroup of tumours that are more frequently encountered in the mesial temporal lobe are the so-called longstanding epilepsy-associated tumours, such a ganglioglioma and dysembryoplastic neuroepithelial tumours (DNET; Figs. 19 and 20) [30]. Nevertheless, even these tumours rarely occur primarily in the hippocampus and are most frequently found in the parahippocampal and lateral occipitotemporal gyrus, at the transition areas from allocortex to neocortex [31]. Ganglioglioma and DNET are found mostly in children and young adults.

Gangliogliomas are located in the cortex or in the cortex and subcortical white matter. The classical presentation of ganglioglioma on MRI, seen in about 40% of cases, consists of a well-defined cystic mass with a mural nodule. A solid mass showing low to intermediate signal intensity on T1W and high signal intensity on T2W images is also not uncommon. Calcifications are present in about 30% of cases. Enhancement of the solid portion of the tumour is variable, ranging from non-enhancement (50% of cases) to ringlike enhancement to homogeneous enhancement. Perilesional edema is absent or limited [30].

On MRI, DNET usually appear as multilobulated or gyriform cysts which are located in the cortex or in the cortex and subcortical white matter. The multilobulated cysts are characteristically hypointense on T1- and strongly hyperintense on T2W images. On FLAIR images, they have mixed signal intensity and often a peripheral rim of high signal intensity, the so-called “bright rim sign”, can be seen. Enhancement is seen in about 30% of cases, calcifications in less than 20%. Rarely, only a solitary large cyst is seen. Diffusion restriction is absent [30].

### Vascular disease

#### Arterial ischemic stroke

**Fig. 21:**
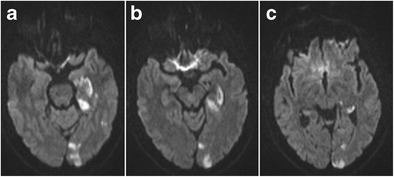
Hippocampal infarction at the level of the longitudinal terminal artery segments in posterior cerebral artery stroke. Axial diffusion-weighted images at the level of the hippocampal head (**a**), body (**b**) and tail (**c**) show diffusion restriction laterally in the hippocampus extending from the body to tail at the level of the longitudinal terminal segments, as well as several cortical diffusion-restrictive foci in the left occipital lobe

**Fig. 22:**
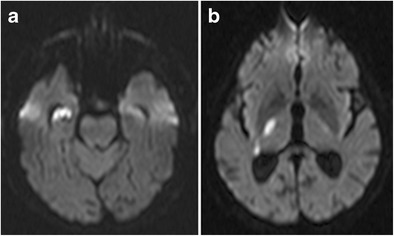
Infarction of the hippocampal head in anterior choroidal artery stroke. Axial diffusion-weighted images at the level of the hippocampal head (**a**) and the basal ganglia (**b**) show diffusion restriction in the hippocampal head and in the posterior limb of the internal capsule

As discussed earlier, the hippocampus has a complex arterial supply with three hippocampal arteries or groups of arteries supplying the hippocampal head, body and tail. The anterior choroidal artery has a variable contribution to the vascularization of the hippocampal head, which may be preponderant in some cases [1]. Hence, the hippocampal head can be involved in anterior choroidal artery infarctions and the entire hippocampus in posterior cerebral artery infarctions (Figs. 21 and 22) [32]. Of note is that infarction limited to the hippocampus is rare and in acute hippocampal infarction, additional extrahippocampal infarctions in the territory of the anterior choroidal artery or posterior cerebral artery are highly likely [31]. This can be used to differentiate acute ischemic hippocampal infarction from other conditions manifesting with hippocampal diffusion restriction, such as transient global amnesia and seizure-induced abnormalities [32].

#### Transient global amnesia

**Fig. 23:**
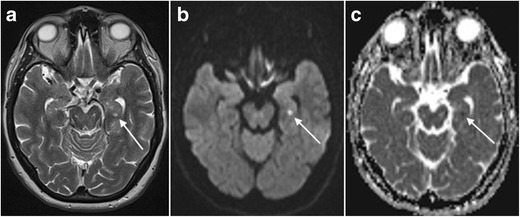
64-year-old man with transient global amnesia. Axial T2W (**a**) and diffusion-weighted images (**b**) with an ADC map (**c**) show a small T2-hyperintense, diffusion-restrictive focus in the left hippocampal body (*white arrows*)

Transient global amnesia is a clinical syndrome characterized by acute retrograde amnesia, generally occurring in patients older than 60 years of age and lasting less than 24 hours. Its aetiology is unclear and several hypotheses have been formulated, including arterial thrombo-embolism, venous congestion, epilepsy and migraine. Although the diagnosis is mainly clinical, the detection of uni- or bilateral small punctate foci of diffusion restriction on MR imaging can help to confirm it. On MRI, hippocampal signal abnormalities can be found in up to 85% of patients, depending on the used MRI parameters and the time elapsed from onset (Fig. 23) [33].

### Toxic-metabolic diseases

**Fig. 24:**
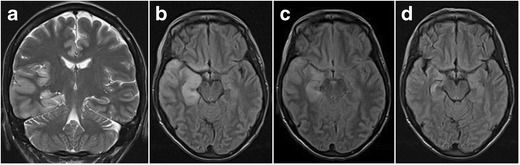
Status epilepticus. A 30-year-old woman with epilepsy quit her anti-epileptic treatment during pregnancy and was brought to the hospital in status epilepticus. Coronal T2W (**a**) and axial FLAIR images (**b**) in the acute phase show extensive cortical oedema in the right temporal lobe with involvement of the right hippocampus as well as in the right insula. There was no diffusion restriction or contrast enhancement (images not shown). Control MRI performed 4 days later after initiation of anti-epileptic treatment shows regression of the oedema on axial FLAIR (**c**). On follow-up MRI performed one month later, axial FLAIR shows volume loss of the right hippocampus as well as increased signal intensity, reflecting secondary gliotic changes (**d**). Alternatively, this could also be a preexistent mesial temporal sclerosis. As extensive clinical work-up revealed no other possible cause for the observed cortical oedema, final diagnosis was seizure-induced cortical edema

**Fig. 25:**
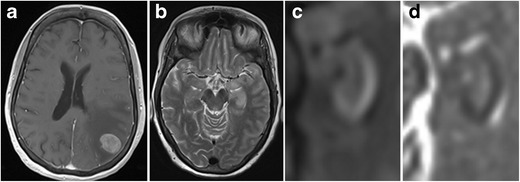
Postictal edema. A 60-year-old woman with stage 4 bronchial carcinoma was admitted to the ER because of generalized epileptic seizures. Contrast-enhanced axial T1 (**a**) showed a contrast-enhancing mass lesion with extensive perilesional oedema in the left parietal lobe, corresponding to a brain metastasis. An axial T2 image (**b**) shows increased signal intensity in the left hippocampus and amygdala. Diffusion-weighted images (**c**) with an ADC map (**d**) show restricted diffusion laterally in the left hippocampus

**Fig. 26:**
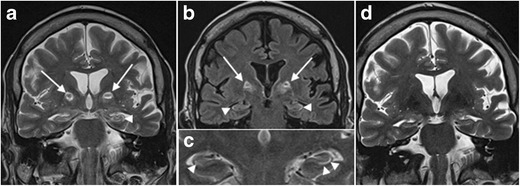
Presumed carbon monoxide poisoning. A 49-year-old man was found comatose and with an alcohol intoxication in his garage next to his car, which was not running. The patient received an MRI 14 days after admittance to the hospital. Coronal T2-weighted (**a,c**) and FLAIR images (**c**) showed T2-hyperintense lesions with central iso- to hypo-intense areas in both pallidi (*white arrows*) as well as T2 hyper-intensity of both hippocampi from head to tail involving the CA2 sector on the right and the CA1 and CA2 sectors on the left (*white arrowheads*). On follow-up MRI performed 4 months later, these abnormalities had disappeared (**d**). The patient suffered from amnesia and the details of what happened exactly remain a mystery to this date. The patient was suicidal and the abnormalities in the pallidi are very suggestive for carbon monoxide poisoning, however, with the hippocampal abnormalities reflecting damage to the especially vulnerable Sommers CA2 sector of the hippocampi

**Fig. 27:**
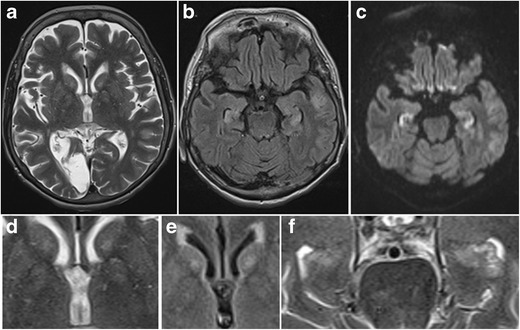
Hypoglycemic encephalopathy. A 60-year-old diabetic woman with an old right occipital infarction tried to commit suicide by overdosing on insulin. MRI performed 7 days after being admitted to the intensive care unit shows subtle patchy increased T2 signal in the bilateral caudate and lentiform nuclei (**a**), which is better appreciated on the zoomed-in axial T2 and FLAIR images (**d, e**). Increased T2-FLAIR signal intensity and restricted diffusion is also observed in both hippocampi (**b, c**). Zoomed-in axial T2 images of the hippocampal head nicely illustrate the increased T2 signal (**f**)

Due to the vulnerability of the hippocampus to excitotoxic brain injury and its primary or secondary involvement in many types of epileptic seizures, the hippocampus is the most frequent location of acute seizure-induced brain abnormalities, which, on MRI, manifest as increased cortical signal intensity on T2W and FLAIR images with or without diffusion restriction (Figs. 24 and 25) [34]. When diffusion restriction is present, it does not automatically imply neuronal cell death, as seizure-induced diffusion-restrictive lesions often show reversibility and are, therefore, sustained by conditions different from ischemic cytotoxic edema [34]. The susceptibility of the hippocampus to excitotoxic or hypoxic brain injury also explains its involvement in many kinds of toxic-metabolic disorders, a detailed description of which is beyond the scope of this review and, as these abnormalities are generally bilateral, have already been described more extensively in pictorial reviews focusing on bilateral temporal lobe disease (Fig. 26 and 27) [35].

## Conclusion

MRI is the preferred imaging technique to assess hippocampal anatomy and pathology. The main indications for tailored depiction of the hippocampus are mesial temporal sclerosis and dementia. A wide range of pathologic conditions with often similar imaging characteristics can be centred in and around the hippocampus, however, and correlation with clinical data is often necessary to come to a correct diagnosis or formulate a sound differential diagnosis.

## Abbreviations

AD: Alzheimer’s dementia
DNET: Dysembryoblastic neuroepithelial tumour
IHI: Incomplete hippocampal inversion
HSE: Herpes simplex encephalitis
LE: Limbic encephalitis
MTA: Mesial temporal atrophy
MTS: Mesial temporal sclerosis
